# Recovery during a crisis: facing the challenges of risk assessment and resilience management of COVID-19

**DOI:** 10.1007/s10669-020-09775-y

**Published:** 2020-05-24

**Authors:** Scira Menoni, Reimund Schwarze

**Affiliations:** 1 DABC, Politecnico di Milano, Milan, Italy; 2 DKKV and European University Viadrina, Frankfurt/O., Germany

**Keywords:** Recovery from measures to mitigate infection spread, Resilience management in case of pandemic, Transfer crisis and resilience management concepts from natural hazards to pandemic, Communication of risk and mitigation measures, Effectiveness of mitigation measures

## Abstract

The paper offers a disaster risk management perspective to analyze the COVID-19 pandemic and to propose and assess non-pharmaceutical mitigation measures for the recovery phase. Three main aspects are tackled: (i) the need to take a scenario-based approach; (i) the need to propose more fine-tuned and context-sensitive mitigation measures, the effectiveness and the cost–benefit of which must be carefully appraised; (iii) better communication as a fundamental pillar of any mitigation measure. Evidence and ideas from the field of natural disasters and man-made technological incidents are applied to tackle the health risk posed by the SARS-COV 2 virus and its rapid spread according to a multi-disciplinary perspective that addresses the health-related challenges and the need to avoid societal and economic breakdown.

## Introduction

The current pandemic crisis, while comparable as a medical challenge, is fundamentally different from any other in the past due to its social, economic and political context. Even the more recent one, the SARS-CoV crisis in 2002–2003, must be considered different because of the incomparably higher contagion rate and the much larger number of goods and people traveling around the world (Zhang et al. 2020).

However, many aspects of the crisis that we are facing have striking similarities with what has been widely discussed in risk management literature for decades, albeit sometimes in other “hazards” domains, and which deserve to be brought to the attention of a larger public and decision makers.

As seekers of information that are not experts in the field of medicine and public health, we recognize a significant gap between the information that has been commendably offered to public access by relevant Journals and Publishers^1^ and the information provided by public authorities in different countries on the measures that have been taken to control the spread of the virus. A quick screening of the scientific literature available on the mentioned COVID-19 platforms shows that the vast majority of articles deal with the technical analysis of the characteristics of the new virus, the challenges in identifying appropriate treatment, in clarifying infection transmission modalities (from asymptomatic individuals for example), while only few address the many practical and psychological challenges for the first “rescuers” that are represented by the medical and hospital personnel in general (Liu et al. 2020; Zhang 2020). Few address the issue of psychological effects of the required quarantine (Brooks et al. 2020), which became a very relevant issue in the pandemic’s unfolding, as it involves millions of people for an unclear period of time. An important distinction that has been made regarding any non-pharmaceutical treatment of the pandemic is between suppression and mitigation measures (Ferguson et al. 16th March 2020). The former corresponds to a lockdown at the regional or country level such as the one initially declared for the Hubei province in China in January and or in Italy on March 8. The latter is a compound of behavioral measures such as hand washing and protective mask wearing, but also selective closure of play grounds, schools and universities, isolation of age groups and individuals more at risk of getting seriously sick and isolation and quarantine for those found positive with symptoms. Now, as the countries that have reached the first peak of the outbreak are taking forward-looking decisions on how to proceed in order not to disrupt the economy even harder and avoid societal breakdown, the focus of research and practice should be on recovery. However, it can be expected that suppression measures will be necessary for a rather long period of time, overlapping with the early recovery phases, to avoid new waves of infection until a safe vaccine will be available in the required quantity, which is expected to be between 12 and 18 months according to Ferguson et al. (2020). While at least 60 laboratories and research centers are currently working on a vaccine, there is no guarantee that an effective one will be actually found at all (Callaway 2020). But even in the case a vaccine will be found, the lock down of entire economies for an unlimited period of time is not an option to be considered under a resilience approach. Massaro et al. (2018) show convincingly that there is a critical threshold at which the risk of infections is decreasing to a desirable level beyond which the societal and economic costs are such that the “deterioration of systems functionality” is unbearable. Resilience management requires instead to “evaluate cross-domain alternatives to identify a policy design that enhances the system’s ability to (i) plan for such adverse events, (ii) absorb stress, (iii) recover, and (iv) predict and prepare for future stressors through necessary adaptation”.

Located between the end of the emergency and advanced reconstruction, recovery from disasters has only recently received increasing scientific attention. Researchers continue to strive for a full understanding of the conditions and nature of the decisions and actions that drive communities either to a successful or, instead, to an unsuccessful recovery and thus to an unsuccessful reconstruction (Chang and Olschansky 2009). Recovery is often characterized by a considerable uncertainty as to how to manage a very large number of problems, while lifting some of the exceptional instruments and extraordinary power that are associated with the state of emergency. As for the current crisis, whilst we were not really able to learn from each other and coordinate for the emergency, we can still try doing this for the recovery, at least putting in place tracking and surveillance mechanisms in a more harmonized fashion and also learn from each other regarding what does and what does not lead to a lead to a successful recovery of society and ways of life.

## From a risk management perspective: planning, learning, scenario-based recovery strategy

The lack of preparedness in facing this global pandemic is rather evident amongcrisis managers and even eminent entrepreneurs, despite several warnings that were out there issued by medical experts. Reports depicting what a likely impact of a widespread coronavirus infection could look like and corresponding countermeasures were previously available in newspapers and regional and national risk assessments in various countries.^2^ Yet this phenomenon of unheard warnings is not new in the field of natural and man-made hazards. One year before Hurricane Katrina provoked one of the largest disasters in the history of the U.S., a scenario exercise was conducted to respond to a simulated hurricane named Pam (Griffin 2007). The exercise elicited many weaknesses and gaps in the preparedness of key organizations, yet little was done to fix them ahead of the incident. We know from other hazard fields that organizations are often reluctant to prepare for extreme or what may look like worst case scenarios as they do not really want to face their own vulnerabilities. Such reluctancy is often hidden behind arguments related to the need to act, based solely on scientific scenarios, to events that are otherwise framed within administrative procedures and bureaucratic approval processes. Any new input is regarded with suspect as it is perceived by involved officials as frustrating their already conducted efforts to plan and prepare. In complete contrast to this, the scenario approach can however be a mean to test and stress organizational response capacity without having to pay the real costs that are entailed by failures and mistakes made in real circumstances, as correctly put by Lagadec (1995).

While several investigation reports unheard warnings can be expected at the end of this emergency, the question now is how to prepare and plan for the next stage, for the recovery, considering that the duration of the hazard could be going to last for long period of time, longer than just 2 weeks or 1 month needed to diminish the first peak of the outbreak, until effective treatment and a vaccine do become available at a worldwide scale. Some initial lessons can however be already learned from the first emergency period as experienced in the last few weeks that should be leveraged on to proceed with the urgently needed recovery. In any such early crises medical advisors are asked to provide recommendations “on the fly”, based on what becomes new or updated information (Zhang et al. 2020). This provides a typical case where a Bayesian type of hazard and risk assessment approach is essential, i.e., a decision-making process being able to assess new information whenever it becomes available (Guagenti and Petrini 2017). The uncertainties involved in such estimates (Viceconte and Petrosillo 2020) are not different from other natural or man-made hazards, e.g., terror- and crime fighting situations, volcanic eruptions or earthquakes, albeit less “global”. In highly uncertain conditions, preventative measures are neither easy to decide nor to communicate. And discussions about such measures are difficult not only with the public but also (mainly) between scientists and decision makers who are not experts in the field of medicine at the offset of a crisis.

Even in these early periods, careful attention should be paid not only to the damage and loss scenarios (that is what may be the potential negative consequences of the virus spread with no control), but also to scenarios produced by alternative response options. Between hand washing, closure strategies and the development of an effective vaccine and treatment that were considered in the early phases of the COVID-19 crisis, there is a wide margin of options that could be and were undertaken by different countries in an uncoordinated fashion. Often so, there seems to be little room for responses which are not on the opposite side of the scale, i.e., which are not only total closure or business as usual. Although more fine-tuned measures are probably harder to implement, they become essential in the recovery phase entirely in the spirit of the statement made by Vale and Campanella (2005): "We may all be made to survive [author's note: with extreme suppression measures], but it takes intelligence and competence to survive well [with graded measures]".

In order to open up the range of alternative options that can be creatively thought of for the recovery, a first issue relates to the kind of scientific advisors that are able to extract in short time lessons from past experience blended with real time acquired information, of course with the necessary caution but also taking the significant risk of saying something that could prove to be false later on (Lagadec 1995). This is a capacity that contrasts with the legal consequences that may be brought later by the same scientists, so sometimes going for caution is a way of escaping future charges. Something that in the more recent decades has become a real issue (Lauta 2016; Benessia and De Marchi 2016). The position of scientists as public advisors and advisors for decision makers has been the object of many studies, related for example to decisions regarding whether to evacuate or not in the face of premonitory signals of a volcanic eruption (Bretton et al. 2015). The finding is that even in ‘early stage’ societal risk governance environment, we need outcome-focused ‘operational forecasting’ (Marzocchi et al. 2012)—not only with regard to spatial and physical parameters of the hazard but including the interaction with civil protection authorities and at-risk communities.

This crisis again proves that a more interdisciplinary approach is necessary to tackle global, systemic, complex issues where there are several ripple effects, backward responsibility, and consequence loops. Such complexity cannot be treated only as a medical affair or as a civil defense problem. Decisions must be clear and coherent, but perhaps better based on information and understanding of the multiple aspects entailed by the crisis environment. A more comprehensive understanding of the medical challenges within a risk management perspective might prove more effective, which implies inviting to the consultancy table a larger variety of experts, including economists, researchers in the risk domain, practitioners and academics, with the mandate to make their knowledge and analytical efforts converge on the framing of both the problems and the solutions. In fact, what should be asked from advisors is not an expert opinion but a truly interdisciplinary effort to develop likely scenarios that will take into consideration the multiple aspects of the hazard (the virus spread), exposure (the various situations and places of high concentration of people), vulnerability (physical and systemic, such as social and economic). Scenarios should be based not on the specific specialties of individual advisors (or not only) but on the capacity to synthetize in a very short time the body of knowledge useful to the case that has been produced in each discipline and in coordination with other disciplines. In addition to scientists and experts, representatives of relevant sections of society, including trade union organizations, representatives of school teachers and university deans, and the business community should be involved in the decision-making process. "Experts" are undoubtedly knowledgeable about technical and scientific aspects, but not about the choice (Calabresi and Bobbitt 1978), which in risk management implies both facts and closely linked values (Beck 1992).

## Assessment of non-pharmaceutical mitigation measures through the lenses of effectiveness, cost–benefit and multicriteria approaches

### Effectiveness of taken measures

An interdisciplinary framing of the problem and the objectives to be achieved with non-pharmaceutical mitigation measures is a key aspect that should be very high in decision makers’ priority. The rationale of measures that have been taken by some countries at the beginning of March may help illustrate the contradictions (apparent or real) that must be learned from (and overcome) in the next weeks and months. A legitimate question could have been raised for example regarding the numbers included in different decrees and ordinances: why in France gatherings of more than 5000 (5th March 2020) people were initially prohibited whilst in Switzerland (28th February 2020) the number was dropping to 1000 and in the USA to 10 people (16th March 2020)? Is it a matter of probability? Arguably, from what can be understood from the mathematical and statistical branch of epidemiological studies, the larger the number of potential contacts the faster and more numerous is the progression of the infection in the community. But then why 5000 and not 1000 or 10? Someone should have clearly explained that this was a purely political decision with no ground in epidemiological studies. Or perhaps not? Certainly, the discrepancy between those numbers raises logical questions in the public. The same goes with the reasoning behind initial attempts to contain the infection. Authorities, based on biomedical and epidemiological studies, should have explained why it made sense to circumscribe zones inside a country (Hubei, 23th January 2020, some municipalities in the Lombardia Region, 22nd February) if the virus has already been circulating around the world since December 2019 most likely. Also, one may have legitimately asked if those measures made sense in a community where a large number of cases with symptoms is backed by an equally large or even much larger number of infected people that do not display symptoms and therefore go unnoticed.

In order to overcome such initial contradictions and lack of coherence among measures, a clear objective must be set now for the recovery plan: opening economic activities and services having in mind the need to keep the total number of infections both globally and in geographic hotspots at a pace that can be tackled by countries health care services. If this objective can be agreed upon, a number of different options can be considered within distinct recovery scenarios.

On the one hand, the buffer capacity of health care facilities and especially intensive care units need to be augmented; however, also finer tuned measures aiming at protecting frontline healthcare workers like doctors and nurses must be put in place. This has to be done not only by providing appropriate sanitary devices but also re-designing hospitals wards, entrances so as to limit the possibility of spreading the infection in emergency posts, in any ward of hospitals that are not devoted to Covid-19. On the other hand, creative solutions for guaranteeing certain levels of physical distancing, hygiene practices must be found in order to permit restarting a significant part of activities and also basic services (including health care services not aimed at treating Covid-19) that have been disrupted or significantly delayed until now. Gradual reopening has to be designed and solutions carefully monitored through clear indicators able to detect as early as possible potential new surges. This requires a rather different system for collecting data related to patients, contagion, with respect to what has already been done.

Somehow this is not very different from what occurs in other disaster fields. The lack of good quality data on post disaster damage and losses for example is a crucial problem that hampers not only the prioritization of needs for the recovery, but also the capacity to learn from the disaster, to ameliorate risk assessment models based on evidence and empirical observation of real events (De Groeve et al. 2013; Marín Ferrer et al. 2016). Although banal for disaster scientists, it must be reminded to experts in other fields, that real events represent scenarios, instances from the wide range of possibilities that can be envisaged. Whilst learning should not be limited to the scenario that has just occurred, failing to do so implies a missed opportunity to widen our knowledge and understanding of implied hazards and risks. As a key component of resilience, the capacity to adapt to a changing environment must be nurtured by data, information and knowledge that can be capitalized on in each event, near miss failure or exercises.

**Fig. 1:**
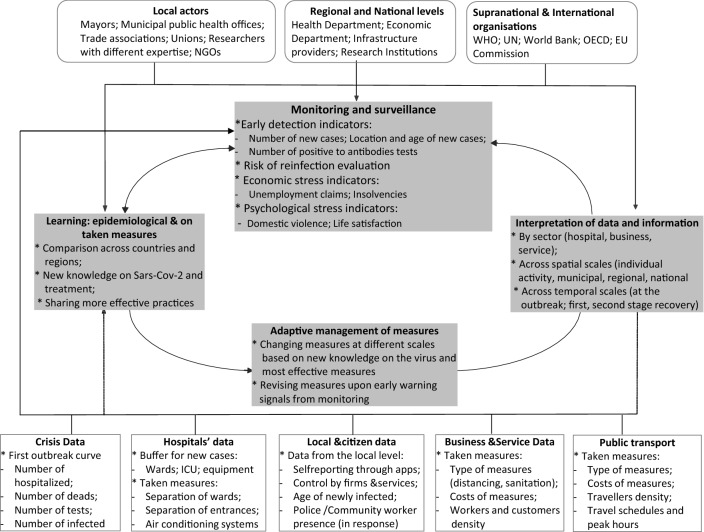
Monitoring and data management as part of adaptive recovery management

Figure 1 sketches how such an adaptive management system should work and adapt fine-tuned decisions regarding operational conditions of economic activities and services, based on a solid data management strategy. As shown in the upper part of the framework international organizations, national and regional governments, local administrations should coordinate to assess the dynamic evolution of risk on the basis of a continuous monitoring mechanism. On the one hand the latter is connected to the lessons learned from the early stages of the emergency and throughout the process of acquiring new information and improved understanding of both the virus spread mechanism and the intrinsic vulnerability of different population groups. On the other hand, such mechanism should be fed by a constant flow of information and data that must be collected according to predefined and carefully selected protocols and filtrated through lenses and criteria that derive from what we know and learn on both the pandemic trend and on the effectiveness of the measures. The effectiveness of measures should be measured not only with respect to the fundamental criteria of keeping the infection at a manageable level but also on the equally important basis of their capacity to allow people to work and carry out activities considering also environmental conditions such as summer heat. Interpretation of data, monitoring and learning are all necessary to support an adaptive management of measures aimed at contrasting the spread of the virus above the sustainable threshold for the health care system. Changes and constant improvement of measures should consider also practices that proved to be effective elsewhere. One important aspect of the framework relates to the spatial and temporal scales at which both monitoring and adaptive management of measures have to be considered. Until now, only national level has been considered in terms of measures’ effectiveness (Flaxman et al. 2020) whilst what is proposed here is an integrated management across levels, from the household to individual businesses to municipal and upward to the regional and national level. Surveillance mechanisms should be developed accordingly, permitting a monitoring of the infection level at a community level rather than regional or national only.

### Costs and benefits of non-pharmaceutical mitigation measures to control the pace of the virus transmission

Typically agencies and persons in charge do not like to defend decisions they have taken, especially during an emergency. This is understandable; however, there are a number of issues to be considered.

First, the idea that highly hierarchical organizations and society work better because nobody questions decisions and everybody does exactly what was ordered has been counteracted by the fact that many times also governments and high in hierarchy organizations do tremendous mistakes, see for example the management of the Fukushima Daichi III nuclear plant accident (The National Diet of Japan 2012). Furthermore, in societies that are less open and democratic hiding of crucial information to get to the truth occurs more frequently (Landemore 2013).

Second, whilst Koopmans (in Zhang et al. 2020) correctly suggests that the current situation is similar to the one faced in front of a flood with the need to protect buildings with sandbags or movable defenses, there are significant differences that must be considered. In the case of floods, means for structural protection are known and (hopefully) available in advance and are somehow considered “positive” even though issues related to upstream consequences arise, for example, when flooding in rural areas is favored to protect cities. Nevertheless, the present situation is more similar to that of hazards requiring mass evacuation of cities, closure of public services and businesses in the face of a potential hazard threat as was the case for the Katrina disaster in New Orleans.

Another important point that puzzles public opinion descends from the difficulties in balancing costs and benefits of restrictions imposed to mitigate the infection spread. In fact, in conditions in which uncertainties and stakes are both high such balance is an extremely sophisticated and complex exercise (Ravetz and Funtowicz 1999). We know this from hundreds of cases in the past in various risk situations and in the face of a multitude of threats to the well-being and security of communities. Uncertainties and stakes unfortunately weigh on both sides of the balance, on both cost and benefit estimation. As for the costs, despite current forecasts of the losses that may be incurred at the global level by economies provided by even eminent organizations (UNCTAD 2020), as researchers in the field we can say that there are significant constraints to the forecasting capacity of underlying models. First because the direct “physical” harm that is generally considered the first link in the cascading effects chain is not likely to be that evident or concentrated in this case. Overall, direct physical damage is what we are best at assessing both pre-end post event. Here the so called “indirect” effects due to the measures put in place to mitigate and/or suppress the spread of the contagion are much more likely to be disruptive and significant. But how bad such effects can be exactly experts cannot really tell. In fact, there is no generally accepted, consistent methodology for estimating the economic impacts of pandemics (Madhav 2017). For example, the majority of models that economists use are designed to depict systems, supply chains under ordinary conditions or under stress that is not single, sudden and rapid onset. How the present crisis will affect the labor market, productivity, etc. are questions difficult to answer, albeit, fundamental to make any real estimate of potential costs of nowadays measures. It must be pointed out also that major public health impacts might be expected as part of indirect and unwanted consequences of the measures. These impacts include pressure and stress on people's mental and general health, exacerbated in contexts where low social protection against unemployment and reduced budget for health care interact through complex loops. Some lessons could be learned from countries that were severely hit by the recent financial crisis (Karanikolos et al. 2013). In a global and networked world, sectors are not only geographically interconnected, but also functionally and systemically. The benefits of the measures are also fraught with uncertainties as briefly discussed above. Therefore, the balance between acceptable costs and reasonably expected benefits is hard to find. There are no exact or optimal solutions here, as only retrospective analysis at the end of the crisis will tell and probably still will be disputable. This explains the large variation of measures between one country and another. Coordination at the international level as called for by many scientists could help, albeit, local application will always require considerations of technical, cultural, social and political feasibility. It may be helpful as well to explain such variations and differences as an inevitable consequence of differences in perspectives, evaluations, and settings. As the crisis is escalating, coordination and collaboration seems much more relevant not only for pandemic management, but also for taking measures and decisions to mitigate the costs of the latter. Collaboration is needed at all scales, ranging from high level head of governments to regional and local level administrations, to share best practices and successful solutions to be experienced during the recovery. One important aspect that has been raised regards the timeliness of action by different organizations. This has been a significant problem in recognizing the threat but also in responding to it and to the challenges different measures imply. As for scientists, researchers in the field of medicine have proved to be fast enough but other fields of studies, including risk management and disaster communities seem to be slower in their reaction, with some exceptions.^3^ Supra-national coordination institutions have been even slower, with the exception of the World Health Organization (WHO)^4^ even though fortunately increasingly active in the second stage of the emergency (see for example Disaster Risk Management Knowledge Center of the EU Commission,^5^ the Organisation for Economic Co-operation and Development,^6^ the United Nations Office for Disaster Risk Reduction^7^).

### Developing advanced non-pharmaceutical mitigation measures for the recovery

In order to move ahead to recovery while preserving the health care capacity of a country and therefore keeping the infection rate below the thresholds above which it becomes unsustainable for the health care system, two convergent lines of action should be implemented, targeting on the one hand services and economic activities, and on the other the monitoring system of the contagion spread.

As for the first, a number of tactics for reopening, i.e., relieving measures for certain age groups or in areas where there are few infected persons, have been already identified and discussed in the media and at political level in different countries (for example in the UK and Italy). In general, however, as outlined above, they lack a systemic perspective, whereas they should become part of a more consistent and comprehensive strategy. The first variable on which to act is time: obviously not every activity and service will be reopened at once. Nevertheless, a clear pathway of reopening should be programmed, also to tailor accordingly measures to sustain those activities that will have to reopen last and perhaps still will suffer significant loss even then (for example tourist activities). Such pathway though should not follow the same logic pursued during the emergency. So not only vital critical necessary activities, but whatever can be reopened safely enough, meaning creating safe distances among customers, users, and workers. The second variable that should be considered relates to the conditions at which each activity and service can be made “safe” in terms of physical distancing. The closure of all but critical activities has been done in some cases (as in Italy) on the basis of statistical classification of activities such as the European NACE. This has created all sort of incongruences and some damage to activities that could have stayed opened if a more fine-tuned classification had been considered. In the recovery there is the need to overcome this rough classification, considering activities on the bases of indicators such as: working conditions, number and concentration of workers and customers in the establishments. For each type of activity some reorganization of work can be defined, such as shifts among workers, allowing them to work partly remotely partly on site, the re-allocation of some equipment and machinery in a way to guarantee the safety distance goal.

As for services, including leisure and cultural activities, booking systems to contain the total number of people by time slots can be thought of and relatively easy to implement through appropriate software and hardware. Clearly, a systemic perspective requires to consider also the interconnection among work and service places and people and in particular the transportation system. Also in this regard, a careful programming of flexible entry and exit hours can be carried out, which in combination with booking systems can significantly reduce the number of people using public transport at the same time. In some cities, an increase in the frequency of service, especially during the busiest hours, will provide an additional buffer to allow safety distances.

A special plan must be put in place for social groups that are particularly vulnerable to SARS-COV-2 such as elderly and immune-depressed. Specific measures should be put in place in those facilities such as hospitals and nursing homes, which tend to host a large number of individuals with these characteristics. However for those who live at home, a plan for meeting with friends and relatives must also be considered so that can lead a more normal life. This is of outmost importance, as the mental health of these social groups is as important as their physical health.

The second line of intervention must address the development of reliable protocols for monitoring and surveillance of the spread of SARS-CoV-2 at a large scale, using a combination of tests for the illness itself and for antibodies. Given the limited comparability of data from different countries on all relevant parameters, from the number of people infected (depending on testing) to the number of deaths (depending on how deaths due to Covid-19 has been accounted for in relation to deaths with different co-morbidities that tested positive for Covid-19), it is unlikely that the indicators to be used for monitoring and surveillance and thresholds to be considered for increasing levels of warning will be the same everywhere.

What has to be put in place in fact is an early warning system that permits to detect rapid increase of infection rate before it becomes uncontrollable. Therefore in a similar vein to other early warning systems that are used for natural hazards, such as volcanoes, for example, a mix of indicators rather than a unique indicator should be considered. Furthermore, such early warning system must be designed for a more local level detection capacity, that is at the level of individual workplaces, services, activities combined with a territorial filter at the municipal, provincial (or county) and regional scales (Martin et al. 2019; Torner et al. 2019; Zemri and Hamdadou 2019).

The monitoring needs to be coupled with a proper treatment of cases found positive to COVID-19 and feedback loops to change practices and measures that are not working according to an early warning strategy similar to the one that exist for some types of natural hazards. In the meantime hygiene practices, including washing hands and sanitizing those surfaces that are more likely to keep the virus for long time should be maintained.

According to an adaptive management of the mitigation measures as depicted in Fig. 1, the first and the second line of interventions should not be considered separately and parallel, but rather in conjunction. On the one hand the measures at workplaces and at service providers (including leisure and cultural services) should be monitored based on careful epidemiological surveillance. On the other, improved therapeutic protocols will permit to avoid overwhelming hospitals and get to the point where Covid-19 is treated as other diseases.

The guidelines detailing the types of measures and actions to be taken will inevitably require revision of some legal norms related to privacy, remote working, etc. that must be design considering as a performance standard to be achieved the maximum level of flexibility as there is the need to change practices as new information and understanding of the spread mechanism become available. The design of norms must be framed within wider consideration of wellness of the communities, respect of crucial democratic and freedom rights as much as possible and embedding the possibility for constant revision and amendment whenever needed. What should be avoided in the recovery is the same separation of interventions that has been witnessed in the emergency. Measures allowing to return to an almost normal life should be coupled with measures to surveille the infection spread and to program better modalities of taking care of patience with a combination of homecare, hospitalization, prevention of the latter that will become possible with the improvement of treatments and a better understanding of the virus impact on humans. Still a fundamental pillar for the full adoption of such new norms and measures relies on a revised and more advanced modality of communicating with the larger public.

## Communication of risk and of non-pharmaceutical mitigation measures

A very wide literature (Perry and Nigg 1985; Glick 2007) is available on past mistakes, good practices, successful and totally wrong information campaigns that it would be impossible to recall them all here. Albeit individual social scientists have been interviewed by media it does not really look like eminent risk communication experts have been involved by decision makers, at least not judging from the issued communication. Some lessons learnt have been adopted, for example in many countries only one authority has been appointed to dispatch the numbers regarding victims, those found to be sick and/or positive to tests but with mild or no symptoms. However, building on established lessons requires to have a wide perspective on many issues and dismantle many myths that still persist in governmental agencies in charge for example of civil protection (Drabek 1986).

It is crucial to maintain a continued positive flow of information. Without this, for example, there may be greater uncertainty, the early lifting of protective measures in the personal environment and consequently new outbreaks may occur. For the acceptance of the communicated messages it is essential that the authorities communicate with the population on an "equal footing". Citizens should be regarded as partners, not as recipients of orders. It is also necessary to communicate with different target groups; different demographic groups must be addressed differently, also multilingual information material should be available (Andrulis et al. 2007; Wong and Sam 2010).

In democratic societies and in the era of internet and social media, where news (fake or true) get “viral” in the web at much higher rates than actual viruses do, there is no way to contain distrust, anger, and fear, sometimes well grounded, if not establishing a mutual confidence and respect relationship between authorities, scientists and the public. This also is the agreed upon result of several studies, projects, and, good practices (De Marchi 2003; Steelman and McCaffrey 2013, see also the EU funded Horizon 2020 Educen project^8^). However, such trustfulness must be built *before* the crisis, during the latter it is harder or even impossible. In contexts where trust and sharing of information is transparent and recognized as such, the provision of information regarding taken decisions must be convincing and based on reasoning and logic. This provision should also consider the inevitable emotional aspects entailed by any emergency condition, not only for the “victims” but also for the decision makers and their consultants.

Authorities are clearly reluctant to share the entire basis of the rationale beyond taken decisions for the fear of “panic”. But here lies one of the toughest contradictions, not new though as perfectly expressed long ago in a book chapter by Handmer (1999). Authorities want the public to be aware of the challenges and agree to follow established rules of conducts and what are generally limitations to their freedom and self-determination. However, they refuse to open the entire evidences on which such decisions have been made based on fear of panic and irrational behaviors, thus, showing the same low level of trust in their citizens that the latter display towards them. An important distinction should be made between “panic” and fear, which is well known in sociology yet not sufficiently acknowledged (Gannt and Gannt 2012). Fear consists in a strong emotional reaction that may be positive because it triggers safeguarding actions (given the information one has). Fear does not necessarily develop into panic, which is negative and to be fought in all possible ways as it paralyzes individuals and impedes them from taking any positive action for their survival. Not letting understandable fear degenerate into panic and anti-social behaviors, which in any case are very uncommon in disasters, depends also on appropriate, consistent, coherent information.

Then the argument goes towards very technical issues that only “experts” can understand. However, the questions that can be asked regarding the measures taken up to now by governments can be answered by unveiling current levels of uncertainties and logical assumptions even without explaining the entire biochemical aspects of super specialized studies, in the same vein as restrictions to visiting a volcano or the determination of mass evacuation can be explained without going into the ultimate detail of the geological, volcanological, geo-chemical, seismological features that drive towards certain albeit changeable decisions. There is no escape to unveiling the body of knowledge, showing that is robust, significantly larger than any time before in history, and in any case the only relevant ground we have and on the other side to admit the contour of uncertainties and ignorance that lead to given decisions only out of precautionary approach that, though, may prove to be key for saving thousands lives.

## Conclusions

In this paper we have attempted to provide a risk management perspective to the pandemic crisis triggered by the spread of SARS-CoV-2 virus, focusing on three main issues. First, a scenario approach should be at the core of recovery, differently from what has occurred in the emergency phase. Second, more advanced, innovative, and fine-tuned mitigation measures should be developed and co-designed with different experts and stakeholders to avoid societal and economic breakdown. Third, improved communication on both the risks associated with the uncontrolled spread of the virus and the measures to slow down the contagion should be encouraged, based on state of the art literature and on best practices in the risk management field.

As for the first issue, it has been suggested that scenarios should be developed jointly by multi-disciplinary teams that should concretely work together to first frame the problem(s) at stake and then develop solutions. The latter consist in much more fine-tuned and context-sensitive mitigation measures that must address the complexity of our societies, the existence of several economic sectors, economic activities, services each requiring the design of appropriate rules of conducts permitting to restart albeit safely. Mitigation measures must be assessed and decided upon based on considerations of effectiveness and cost benefit. Health criteria are key, but they must be balanced against the need to recover in all sectors of human and collective life, to avoid societal and economic breakdown and considering the multiple loops that exist between community well-being and health. Such multiple loops and retroactive feedbacks must be properly addressed in the design of both measures and monitoring protocols to make sure to avoid second and third surges of COVID-19 that are beyond the capacity of the health care system to sustain.

This paper clearly does not have the ambition to be a full research paper but has been developed believing that science and research should in some occasions, if appropriate, provide guidance and advice based on the best available knowledge to address significant societal challenges even at the cost of being inaccurate. In future, there will be the time and the need for more in-depth analysis, for studies on the failures of the response system, but also for a better identification of strategic research areas, including more compelling institutions and practices of truly interdisciplinary research, especially at the frontier of today's disciplines, both theoretical and applied.

## Footnotes

1 See for example https://www.thelancet.com/coronavirus; https://www.elsevier.com/connect/coronavirus-information-center; https://www.cell.com/2019-nCOV

2 The article by Isaac Scher “An internal Pentagon plan from 2017 warned of a coronavirus outbreak – and a massive shortage of medical equipment” published in Business Insider, April 6 2020 (see US Northern Command 2017) and the article written by Andrea Sparaciari “Il disastro coronavirus in Lombardia era già scritto in un audit del 2010, mai applicato”, published in Business Insider Italy, April 17 2020 (See Regione Lombardia 2010). The national risk assessment of Germany of 2012 predicted the course of a SARS-CoV pandemic in good agreement with current events, however with a 3-year period for vaccine development and as many as 7,5 million deaths (Deutscher Bundestag 2013).

3 See for example https://www.theisrm.org/en/nova-coronavirus-lessons-learned-from-a-crisis-management-perspective and https://steps-centre.org/blog/postnormal-pandemics-why-covid-19-requires-a-new-approach-to-science/

4 https://www.who.int/emergencies/diseases/novel-coronavirus-2019/global-research-on-novel-coronavirus-2019-ncov

5 https://drmkc.jrc.ec.europa.eu/overview/COVID-19

6 https://www.oecd.org/coronavirus/en/

7 https://www.undrr.org/drr-and-covid-19;

8 https://educenhandbook.eu/
